# Supplementary figures and images for: Neuroblastoma Cells Depend on CSB for Faithful Execution of Cytokinesis and Survival
Source: Int J Mol Sci. 2021 Sep 17;22(18):10070. doi: 10.3390/ijms221810070 (PMC8465547; doi:10.3390/ijms221810070)

Figure S1

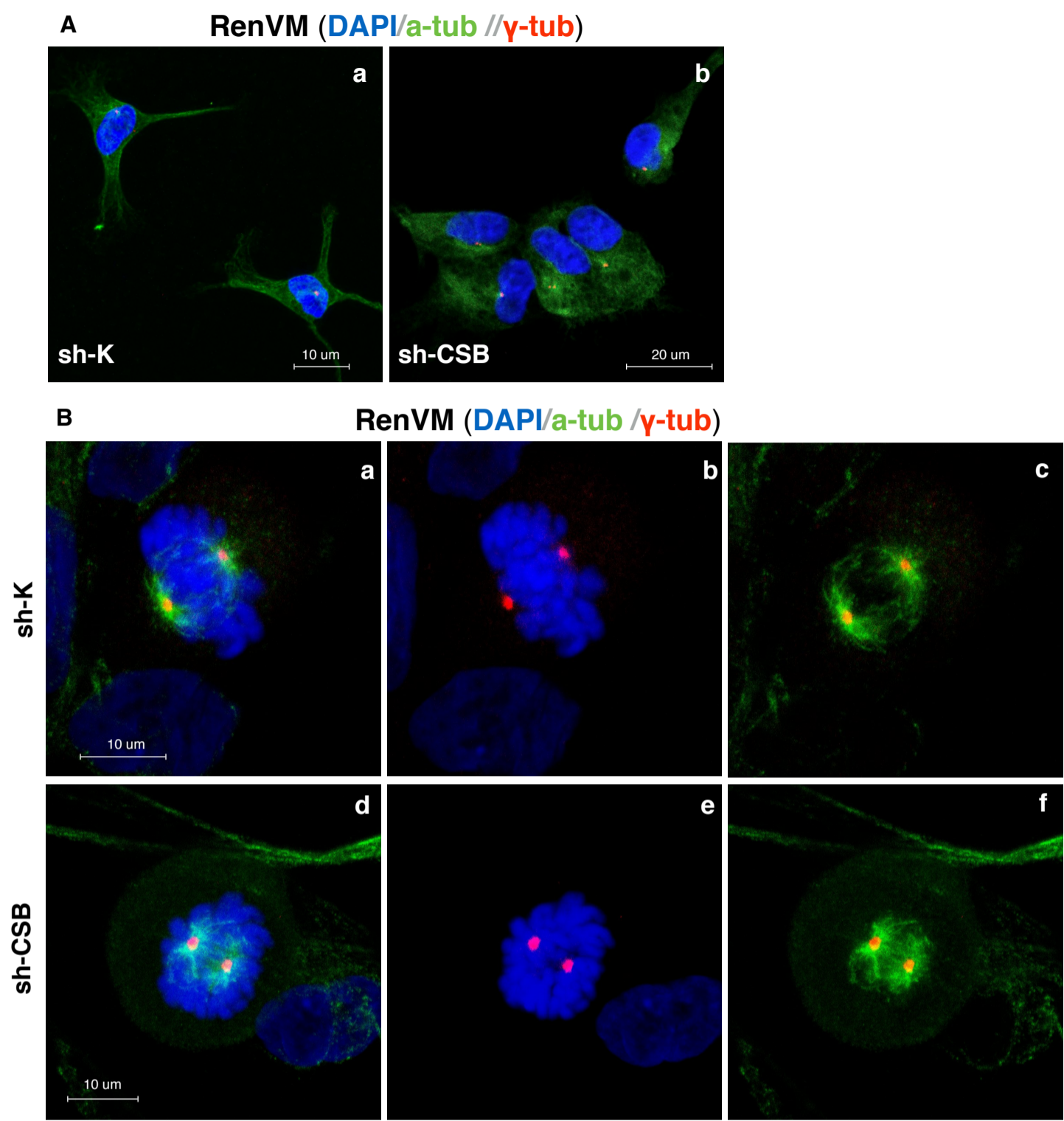

Supplement: Supplementary file 1 [file ijms-22-10070-s001.zip › ijms-1349229-supplementary.pdf]
